# Nanocavities for Molecular Optomechanics: Their Fundamental Description and Applications

**DOI:** 10.1021/acsphotonics.4c01548

**Published:** 2024-11-06

**Authors:** Philippe Roelli, Huatian Hu, Ewold Verhagen, Stephanie Reich, Christophe Galland

**Affiliations:** † Nano-optics Group, CIC nanoGUNE BRTA, E-20018 Donostia-San Sebastián, Spain; ‡ Center for Biomolecular Nanotechnologies, Istituto Italiano di Tecnologia, via Barsanti 14, Arnesano, 73010, Italy; § Center for Nanophotonics, NWO Institute AMOLF, Science Park 104, 1098 XG Amsterdam, The Netherlands; ∥ Department of Physics, Freie Universität Berlin, 14195 Berlin, Germany; ⊥ Institute of Physics, Swiss Federal Institute of Technology Lausanne (EPFL), CH-1015 Lausanne, Switzerland; # Center of Quantum Science and Engineering, Swiss Federal Institute of Technology Lausanne (EPFL), CH-1015 Lausanne, Switzerland

**Keywords:** Plasmonic antennas, Surface-enhanced Raman scattering, Cavity optomechanics, Molecular vibrations, Raman spectroscopy

## Abstract

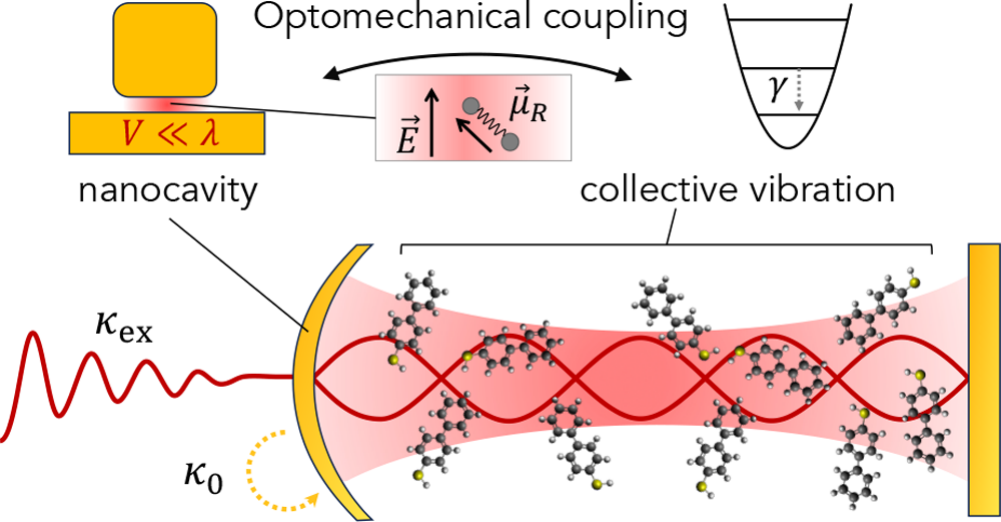

Vibrational Raman scattering—a process where light exchanges energy with a molecular vibration through inelastic scattering—is most fundamentally described in a quantum framework where both light and vibration are quantized. When the Raman scatterer is embedded inside a plasmonic nanocavity, as in some sufficiently controlled implementations of surface-enhanced Raman scattering (SERS), the coupled system realizes an optomechanical cavity where coherent and parametrically amplified light–vibration interaction becomes a resource for vibrational state engineering and nanoscale nonlinear optics. The purpose of this Perspective is to clarify the connection between the languages and parameters used in the fields of molecular cavity optomechanics (McOM) versus its conventional, “macroscopic” counterpart and to summarize the main results achieved so far in McOM and the most pressing experimental and theoretical challenges. We aim to make the theoretical framework of molecular cavity optomechanics practically usable for the SERS and nanoplasmonics community at large. While quality factors (*Q*) and mode volumes (*V*) essentially describe the performance of a nanocavity in enhancing light-matter interaction, we point to the light-cavity coupling efficiencies (η) and optomechanical cooperativities () as the key parameters for molecular optomechanics. As an illustration of the significance of these quantities, we investigate the feasibility of observing optomechanically induced transparency with a molecular vibration—a measurement that would allow for a direct estimate of the optomechanical cooperativity.

## Introduction

Molecular cavity optomechanics (McOM) aims at unifying under a common theoretical description and a common language two distinct research areas: surface- and tip-enhanced Raman scattering (SERS and TERS) on the one hand and (macroscopic) cavity optomechanics on the other hand. SERS and TERS were mostly developed by chemists, spectroscopists, and surface scientists; they describe the huge enhancement of vibrational Raman scattering intensity from molecules interacting with the near field of metallic nanostructures supporting localized surface plasmon polaritons.^1^ In contrast, cavity optomechanics studies the coherent interaction (down to the quantum regime) between light trapped in a dielectric cavity and a mechanical oscillator^2^ whose mass may range from an atomically thin membrane^3^ to a kilogram-scale suspended mirror.^4^

First released in ref (5) and initially motivated by the experimental observation of anomalously narrow SERS enhancement profiles under laser wavelength scans in ref (6), this connection prompted the development of full quantum models for SERS and TERS that account for the different forms of backaction imparted on the molecular vibration by the laser-driven plasmonic resonance.^5,7−11^ McOM has been fuelling inspiration for new conceptual and experimental developments in the field of SERS and TERS, where the focus is not on chemical or material analysis, but rather on achieving classical and quantum control of molecular vibrations and developing new nanoscale optical devices leveraging optomechanical nonlinearities.^12^ Progress in McOM hinges on two pillars: (i) a proper understanding of the theoretical framework and its connection with designed and measured nanocavity parameters, and (ii) an improved control and power resilience of device parameters governing light-nanocavity and nanocavity-vibration couplings, supplemented with critical attention to other sources of nonlinearities that may interfere with optomechanical phenomena. Mastering these two pillars will allow the engineering of new nanocavities and molecules that feature improved stability and finer control over the plasmon-molecule coupling and dissipation rates, with the overarching goal of reaching large optomechanical cooperativities () and nanocavity coupling efficiencies close to unity–which has become routine for microfabricated oscillators coupled to dielectric cavities.^2^

These two pillars are discussed in this Perspective after a brief reminder of the conventional theoretical description of SERS. In this Perspective, we place special emphasis on the description of light-nanocavity coupling and aim to clarify its connection with the input-output formalism used in cavity optomechanics. We will therefore present a more complete analogy between a typical SERS experiment on a plasmonic nanocavity and a macroscopic cavity optomechanical setup, highlighting the specificities of McOM. We also propose a scheme to perform optomechanically induced transparency measurement in McOM, a technique that would provide direct access to the optomechanical cooperativity. We conclude with an overview of some of the most appealing perspectives in the field.

## Basics of Vibrational Raman Scattering and SERS Enhancement Mechanisms

Before describing the theoretical and experimental aspects of McOM, we briefly review the broadly accepted concepts underlying SERS and TERS. The Raman effect^1^ is first described in a classical model: a molecule under the applied monochromatic field **E** oscillating at frequency ω_*L*_ will experience an induced dipole **μ**(*t*) = **α**(*t*) · **E** cos ω_*L*_*t* where **α**(*t*) ≃ **α**_0_ + **α**_*R*_ cos Ω_ν_*t* is the 3-dimensional second rank polarizability tensor that is modulated by the vibration. For simplicity, we will consider here only a single normal mode with vibrational frequency Ω_ν_ ≪ ω_*L*_ (vibrational frequencies are in the range of 1–100 THz while laser frequencies are typically above 500 THz) and associated normal mode coordinate *Q*_ν_. This mode is Raman active if ; in this case, for appropriate orientation of the incoming field, the Raman dipole  radiates at two new frequencies ω_*S*_ = ω_*L*_ – Ω_ν_ (Stokes) and ω_*aS*_ = ω_*L*_ + Ω_ν_ (anti-Stokes), which constitute the inelastic Raman scattered field. Note that the classical model predicts Stokes and anti-Stokes sidebands of equal amplitudes; a semiclassical model where the vibration is quantized correctly predicts the observed asymmetry.^1,13^

SERS was discovered in the 1970s as molecules absorbed on metal electrodes and other rough metal surfaces were studied with Raman spectroscopy.^14−16^ The scattering intensity from molecules showed a great enhancement, which was soon realized to be a genuine increase in the scattering cross section per molecule and not simply due to the aggregation of molecules on the metal surface. The main enhancement mechanism is called electromagnetic enhancement, arising from the optical near fields of localized surface plasmons close to a metal nanostructure.^1,17,18^ A second enhancement mechanism arises from the chemical interaction between molecule and metal. For example, charge transfer may change the electronic configuration and even vibrational frequencies of the molecules and affect their Raman cross sections. Within McOM, the chemical enhancement has to be included ad hoc in the value of **α**_*R*_ for the molecules. We will thus not discuss it further below, even though it impacts experiments where molecules are directly bound to the metal.^19−21^

When the near field of a localized surface plasmon exceeds the incident field at frequency ω by an enhancement factor *K*(ω), it can be shown that the probability of generating the Raman dipole is enhanced by *K*^2^(ω_*L*_) and that of radiating the scattered light into the far-field by approximately *K*^2^(ω_*S*_).^22^ For a single molecule, the SERS intensity compared to that of Raman scattering in free space is therefore multiplied by the electromagnetic enhancement factor
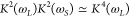
where the last approximation is valid when Ω_ν_ is not too large compared to the plasmonic resonance bandwidth (corresponding to the unresolved sideband regime of McOM).

The strength of this approach is that the enhancement factor *K*(ω) can be calculated by solving Maxwell’s equations only. For well-defined nanostructures with voids or cavities of 1–10 nm dimension, a typical near-field enhancement is *K* ≈ 10^2^, resulting in ∼10^8^ electromagnetic SERS enhancement factors for a probe inside the hot spot. The spatial distribution of plasmonic hotspots, as well as the dependence of the enhancement on polarization and scatterer orientation are predicted exceptionally well within this framework.^23^ However, it has been pointed out that this approach may significantly underestimate the absolute enhancement factors and overestimate the SERS resonance bandwidth in some experiments.^24^

In the microscopic, semiclassical theory of Raman scattering, SERS is viewed as a higher-order Raman process (HoRa),^25^ which is particularly instructive as a similar point of view is taken in McOM. The microscopic theory treats the normal Raman effect within perturbation theory, where a molecule is first excited from its ground state to an excited electronic state and reaches back to the electronic ground state but in an excited vibrational state. The difference between the two transition energies is equal to the energy of the vibration *ℏ*Ω_ν_. SERS is modeled by adding two interaction steps that describe molecular excitation and emission through the localized surface plasmon of resonance frequency ω_*p*_ and line width κ. Two resonances occur when the incoming *ℏω*_*L*_ or scattered *ℏω*_*s*_ = *ℏω*_*L*_ – *ℏ*Ω_ν_ photon energy matches the plasmon energy. A coupling factor describes the plasmon-related interaction in the SERS process, as detailed in ref (25). Note that this framework does not keep track of possible changes in vibrational population and dynamics that could be induced by Raman scattering, which is a gap that the McOM framework fills.

## Optomechanical Model of Nanocavity-Enhanced Raman Scattering

**Figure 1:**
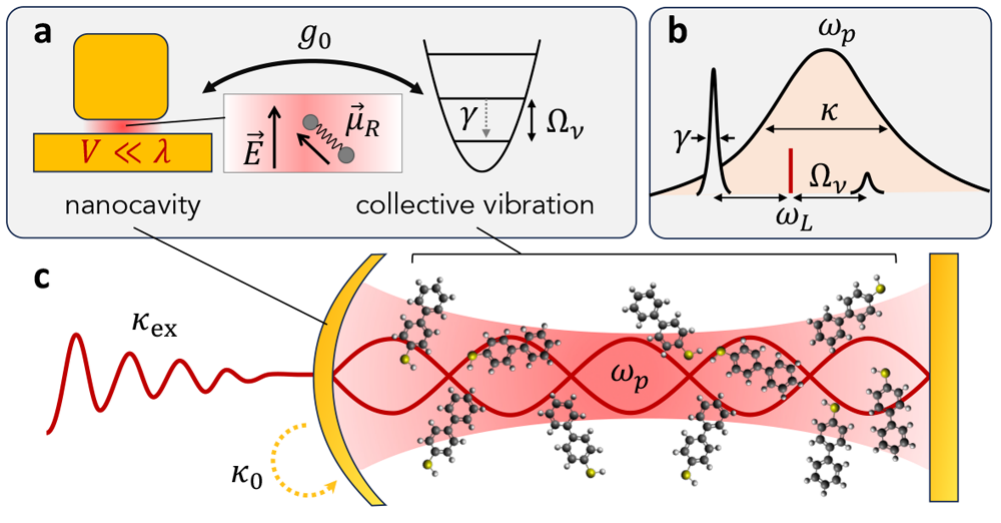
Simplified setting for single-mode molecular cavity optomechanics (McOM). (a) A plasmonic cavity (here sketched as a metallic dimer) supports a plasmonic resonance with radiative and nonradiative decay rates κ_ex_ and κ_0_, respectively. The surface-enhanced local field ***E***(*t*) couples to the induced Raman dipole **μ**_*R*_(*t*) of the vibrating molecule, resulting in the vacuum optomechanical coupling rate *g*_0_. The molecular vibration is damped at a rate γ. (b) Frequency domain schematic showing the plasmonic resonance at frequency ω_*p*_ of width κ = κ_ex_ + κ_0_. Under a single-frequency pump at ω_*L*_, the optomechanical coupling gives rise to two Raman sidebands at ω_*L*_ ± Ω_ν_, where Ω_ν_ is the molecular vibration frequency. Panel (c) is inspired from ref (26).

Molecular cavity optomechanics (McOM) is based on a quantum model of plasmon-enhanced Raman scattering; in a minimal setting one molecular vibrational mode, treated as a harmonic oscillator with resonance frequency Ω_ν_ and decay rate γ, modulates the optical response of the nanocavity characterized by one plasmonic mode with resonance ω_*p*_ ≫ Ω_ν_ and total decay rate κ ≫ γ (this dissipation hierarchy is reversed in Raman lasers based on high-*Q* dielectric cavities^27^ where κ ≪ γ, the rest of the physics being otherwise similar), see Figure 1. Both vibrational and plasmonic degrees-of-freedom obey boson statistics and are supposed to be exchanging energy quanta at rates κ and γ with a thermal bath. The resolved-sideband regime is achieved when Ω_ν_ > κ/2 (Figure 1b), which is possible in McOM despite the fast plasmon decay thanks to the often very high vibrational frequencies (tens of THz). For example, for a *Q*-factor of 10 at 700 nm, the minimum vibrational frequency to be sideband-resolved is 715 cm^–1^ or 21.4 THz.

Pure dephasing is not observed to play a significant role in plasmonic decoherence.^28^ For vibrational modes of molecules, experimental data obtained using 2D IR spectroscopy on monolayers suggest that inhomogeneous broadening strictly dominates over population relaxation and single-molecule pure dephasing rates.^29−33^ For configurations typically studied in SERS, the inhomogeneous (collective) relaxation rate was recently measured to be subpicosecond,^34^ consistent with earlier vibrational sum-frequency studies on monolayers.^35,36^ In the single-molecule limit, a report from 2014 inferred much longer-lived coherence between two vibrational modes of the same molecule, in excess of 10 ps.^37^ If such a result were to be confirmed, it would imply that single-molecule optomechanics, as achieved in the regime of picocavities^38^ or in TERS,^39^ may benefit from reduced vibrational decay rate γ and a corresponding increase in the cooperativity (introduced below).

The single-photon optomechanical coupling rate *g*_0_ is proportional to  with *V* the plasmonic mode volume and **u** the near field polarization vector. Note that the term in parentheses is proportional to the square root of the Raman cross-section of the molecule.^5^ There are two main interpretations for the meaning of *g*_0_: (i) Conventionally, *g*_0_/2π measures the plasmonic resonance frequency shift induced by a vibrational mode displacement whose magnitude equals that of the ground state fluctuations (or zero-point motion);^2,5^ (ii) Alternatively, from a microscopic point of view the interaction energy *ℏg*_0_ relates to the interaction energy  between the local vacuum electric field **E** and the induced Raman dipole **μ**_*R*_ (Figure 1a).^8^

For weak single-photon optomechanical coupling (*g*_0_ ≪ κ, as is the case in all realizations of McOM to date) the interaction Hamiltonian can be linearized. The vibrational and plasmonic modes coherently exchange quanta at a rate , where *N* is the effective number of coupled molecules and *n*_*p*_ the time-averaged occupancy of the plasmonic mode (intracavity plasmon number), which depends on the input laser power *P*_*L*_ and the overall incoupling efficiency and incoupling rate (see below). As a result, for weak coupling Ω ≪ κ, the mechanical vibrations are coupled to the optical bath to which the plasmon mode decays at rate Γ_opt_ ∝ 4*n*_*p*_*Ng*_0_^2^/κ, scaling linearly with both the number of molecules and the number of intracavity plasmons. As the single most important parameter characterizing the performance of an optomechanical cavity, the cooperativity is defined as , which measures the ratio of Γ_opt_ to the decay rate of the vibration. Here,  is known as the single-photon cooperativity.

**Figure 2:**
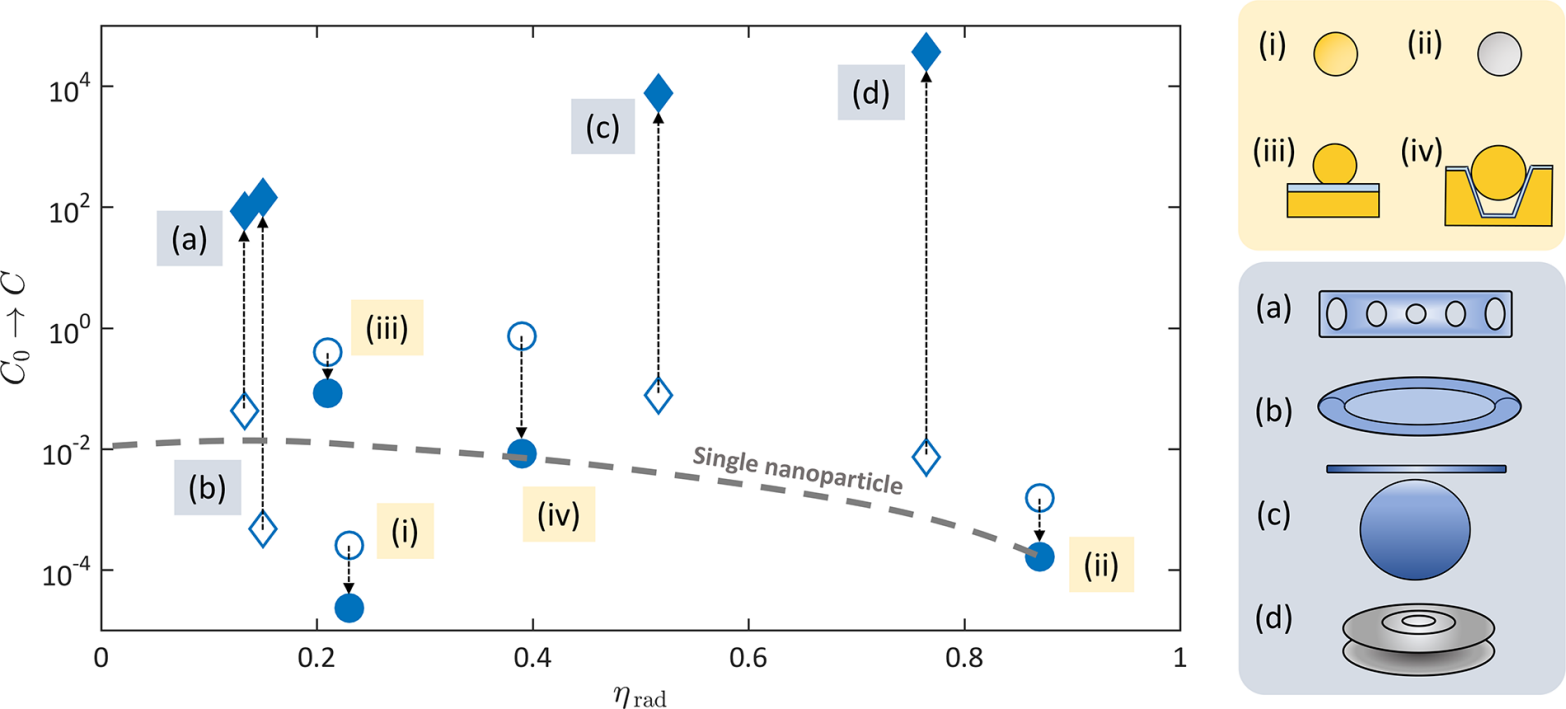
Overview of representative plasmonic (i–iv) and dielectric (a–d) optomechanical cavities in terms of cooperativity and radiative coupling efficiency (defined in Figure 3). For plasmonic cavities, we assume that a monolayer of biphenyl–thiol molecules covers the nanoparticle or the substrate and that the incoming laser power is set as 100 μW in a diffraction limited spot. Empty symbols represent the single-photon cooperativity , while full blue symbols represent . The data for dielectric cavities are compiled from published experiments: (a) ref (41), (b) ref (42), (c) ref (43), and (d) ref (44).

**Figure 3:**
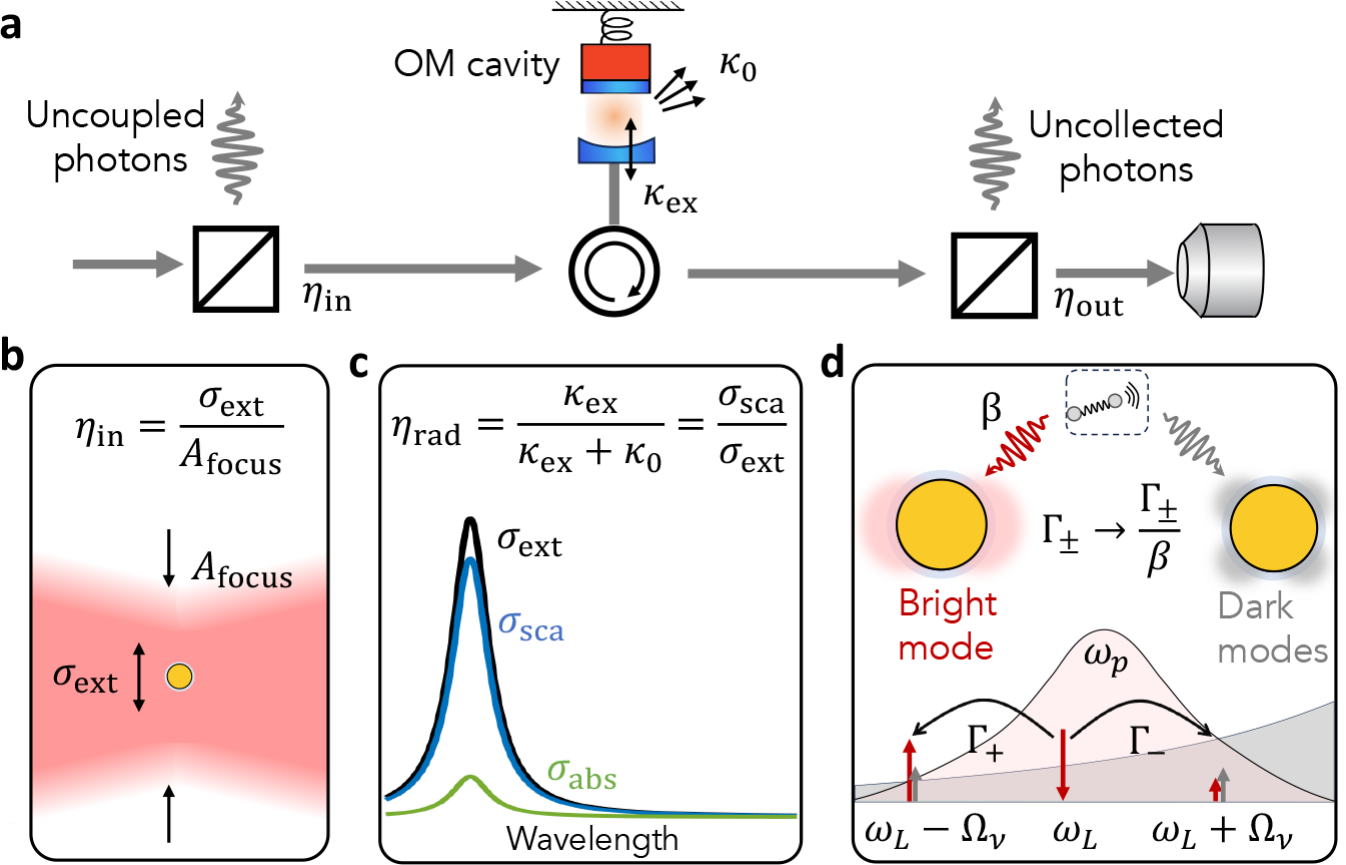
Correspondence between macroscopic and molecular cavity optomechanics. (a) Sketch of general cavity optomechanics scenario where all input and output coupling losses can be modeled by beam splitters with transmissions η_in_, η_out_, respectively; the total decay rate of the cavity is the sum of an external decay rate κ_ex_ allowing for photon exchange with radiation modes and an internal decay rate κ_0_ that accounts for absorption and scattering losses. (b) In McOM, the input coupling efficiency corresponds to the ratio of focal spot size to the extinction cross section of the nanocavity (pictured here as a simple metal nanoparticle coated with molecules). (c) The external coupling efficiency is given by the ratio of scattering to extinction cross section in the single mode limit. (d) The optomechanical coupling occurs in the near field through the Raman polarizability, leading to scattering rates Γ_+,–_, respectively, for Stokes (add a quantum of vibration) and anti-Stokes (removes one) processes. The existence of a spectrally overlapping quasi-continuum of dark modes is responsible for additional Raman scattering contributions in the near field that are, however, not detected in the far field (quenching).

Achieving  is a prerequisite for realizing key applications of cavity optomechanics. As a first example, in the resolved-sideband limit, a laser drive optimally blue-detuned from the cavity resonance modifies the damping rate of the vibration to , causing a line width reduction and coherent amplitude amplification of the vibrational mode^2^ (cold damping, or cooling, is possible under red-detuned drive). More generally,  marks the onset of strongly nonlinear dynamics. As a second example, when using an optomechanical system to implement coherent optical frequency conversion^40^ the efficiency scales as , where  represents the cooperativity computed for the other cavity resonance involved. A necessary condition for optimal conversion is therefore . Figure 2 presents how different nanocavity geometries compare with typical dielectric cavities used in optomechanics in terms of cooperativity and radiative coupling efficiency η_rad_, the latter being a mode-specific property of the nanocavity quantifying its ability to couple to far-field radiation (see definition in Figure 3c). Here one recognizes that, while McOM nanocavities can naturally feature single-photon cooperativities that exceed those of state-of-art dielectric optomechanical resonators, enhancing the coupling through large cavity occupancies is more challenging.

The McOM theory was not initially proposed as a new fundamental form of plasmon-vibration interaction nor a substitute to the accepted SERS enhancement^45^ mechanisms reviewed above; it was shown to recover the same predictions as the electromagnetic theory based on field enhancement in the regime where .^5^ However, a modified and nonlinear response of the cavity and molecular vibration is predicted when  approaches unity. Very strong pumping is required for reaching appreciable values of *n*_*p*_ (recall that ), under which nanocavities can be irreversibly damaged or display other nonlinearities, challenging the experimental realization of this regime.^46−51^ At the same time, since the thermal phonon occupancy *n*_th_ is very low due to the high vibrational frequencies, the cooperativity is the same as the quantum cooperativity (defined as ) in McOM systems, in contrast to most other optomechanical cavities (especially at room temperature) where the quantum cooperativity is much smaller. Moreover, McOM is a quantum formalism that treats vibrational and plasmonic degrees of freedom at the same level of description, with some predictions that go beyond the conventional theory of SERS. Here is a nonexhaustive list of physical phenomena and observables that the McOM formalism predicts:^10^

- In the sideband-resolved regime (κ < 2Ω_ν_) and for blue-detuned pumping, a new enhancement mechanism may be achieved through dynamical backaction,^5^ akin to phonon lasing.
- Quanta of molecular vibration mediate quantum correlations between Raman scattered fields^52^ – as already observed in the absence of a nanocavity;^53−57^
- The collective nature of SERS is important even in the spontaneous scattering regime, in the sense that the Raman scattered field is correlated with a collective quantum of vibration that is coherently shared among all molecules coupled to the nanocavity field^5^ – an effect also observable in the absence of nanocavity through spatial mode filtering.^58^ Cooperative effects among vibrating molecules are expected under sufficiently large cooperativity;^59^
- Vibrational pumping^38,45,47,60−68^ can be understood as the manifestation of quantum backaction,^38^ otherwise difficult to observe in macroscopic cavity optomechanics. It occurs also for resonant cavity pumping and in the unresolved-sideband regime, as soon as . Note that charge transfer between the metal and the molecule was also proposed as a mechanism for vibrational pumping,^69−71^ a process beyond the scope of McOM.
- McOM provides a rigorous framework to study how the multimode nature of plasmonic or hybrid plasmonic-dielectric^72,73^ resonators impacts the SERS signal.^74,75^ For example, multimode McOM predicts a much larger optical spring effect (shift of the Raman peak) with respect to the optomechanical damping rate (change in its line width), which results from the large real part of the dyadic Green’s function in plasmonic nanocavities, itself a consequence of the quasi-continuum of plasmonic modes supported at shorter wavelengths in metallic nanogaps;^75^
- Vibrational sum- and difference-frequency generation can be computed with a proper account of quantum and backaction noise,^76^ with applications in coherent mid-infrared and THz frequency conversion^12,76^ and in vibrational spectroscopy.^77−82^

## Scaling Laws

The performance of a nanocavity in enhancing light-matter interaction in its near field is well characterized by the quality factor *Q* and mode volume *V* (which are modal quantities).^83^ For a single molecule acting as mechanical oscillator in McOM, it has been shown that the single-photon cooperativity  scales as *Q*/*V*^2^ (instead of *Q*/*V* for the Purcell factor).^5^ It is because the field intensity enhancement factor *K*^2^ introduced above scales as 1/*V* and the SERS intensity scales as *K*^4^. In contrast with cavity QED, the OM coupling rate is field-enhanced: If we account for the fact that the intracavity plasmon occupancy *n*_*p*_ scales with *Q* (if the coupling efficiency remains fixed) we find that  scales as (*Q*/*V*)^2^ for a single molecule. In practice, however, *n*_*p*_ is often limited by material failure and optically induced instabilities.

Most experiments deal with ensembles of *N* molecules collectively coupled to the nanocavity mode, resulting in a factor *N* increase in .^5,59^ In the limit where molecules fill the entire mode volume, i.e. when *N* ∝ *V*, we obtain the scaling . As a side note, for a resonant cavity-emitter interaction like in infrared absorption, the collective cooperativity is independent of mode volume, it only depends on the emitter concentration per unit volume.^76^

Unless the single-photon strong coupling regime is reached (*g*_0_ ∼ κ), the parameter of relevance for McOM is not so much  as ; we must therefore precisely estimate the intracavity plasmon number *n*_*p*_, which depends on the far-field to near-field coupling efficiency^84−86^ and on the decomposition of the total plasmon decay rate κ into radiative and nonradiative channels,^87−89^ as is discussed in the next section. The various decay channels are also responsible for the existence of both dispersive and dissipative optomechanical coupling mechanisms, the latter contribution being much less understood while possibly dominant in some McOM realizations^90^ (see below). We remark in passing that if the intracavity plasmon number *n*_*p*_ could be made to increase linearly with mode volume, i.e. the electromagnetic energy density inside the nanocavity would be kept constant, then  would become independent of mode volume. But in practice the highest tolerable laser power is not constrained by mode volume alone and reducing the mode volume is a key pursuit to increase  in McOM.

## Input–Output Formalism

In order to take advantage of the vast body of knowledge in macroscopic cavity optomechanics^2^ to design and interpret McOM experiments, and to gain a physical intuition, we advocate for a proper use of the input-output formalism^91^ in the context of plasmonic nanoantennas and nanocavities.^92^Figure 3 proposes a one-to-one correspondence between the canonical optomechanical cavity framework and that of McOM. We will analyze the situation in terms of photon flux entering and leaving the cavity, from the laser source to the detector. First, if we call Φ_*L*_ the photon flux impinging from the laser on the nanoantenna (here pictured as a simple metallic nanoparticle for simplicity), only a fraction Φ_in_ = η_in_Φ_*L*_ will actually interact with it. The factor η_in_ quantifies the input coupling efficiency: in macroscopic cOM it can account for fiber-to-waveguide coupling losses, for example; in McOM it typically accounts for the overlap of the incident wavefront and the time-reversed radiation field of the nanoantenna, and thereby for the ratio of the extinction cross-section of the nanoantenna σ_ext_ to the laser spot size *A*_focus_ (Figure 3b). Note that for elongated nanorods, nanoparticle-on-mirror cavities and all other structures supporting several modes, the polarization of the incoming field at the position of the nanocavity must be taken into account to estimate η_in_.^86^ A particularity of nanocavities is the difficulty of achieving η_in_ ∼ 1, compared to macroscopic dielectric cavities.

In general, the input mode is populated by a coherent laser field plus the irreducible quantum fluctuations (shot noise); its amplitude couples to the cavity at rate , which for a simple Fabry–Perot cavity is controlled by the transmission of the input mirror and the round-trip time in the cavity. For a plasmonic nanocavity, κ_ex_ measures the rate of radiative energy decay into the far field over the entire solid angle. By reciprocity, κ_ex_ is therefore a measure of how efficiently an external field may excite a particular nanocavity mode (the subscript “ex” stands for “external”). Beside this useful cavity loss channel (in the sense that it connects the cavity to the experimentally controlled fields) other loss channels are captured by κ_0_, including absorption in the metal or excitation of propagating surface plasmon polaritons eventually damped in the metal (for nanocavities built on metallic films). The equations of motion therefore include the vacuum quantum noise entering the cavity through these undesired channels, maintaining the fundamental connection between dissipation and fluctuation.

The total decay rate of the cavity is κ = κ_ex_ + κ_0_, related to the quality factor by *Q* = ω_*p*_/κ. The quality factor alone hides the information on the relative magnitudes of κ_ex_ and κ_0_. For most applications in cavity optomechanics, it is beneficial to work with a critically or overcoupled nanocavity mode, i.e. having κ_ex_ ≥ κ_0_. If we define the external coupling efficiency of a nanocavity mode as η_rad_ = κ_ex_/κ, then the aim is to reach η_rad_ ≥ 0.5. For example, the efficiency of optomechanical frequency conversion is proportional to the products of η_rad_’s at the two frequencies involved, so each η_rad_ must be made close to unity.^40^ In terms of the scattering cross section σ_sca_ and extinction cross section σ_ext_ that are usually computed or measured for plasmonic antennas, we simply have η_rad_ = σ_sca_/σ_ext_, i.e., the nanoantenna albedo (see Figure 3c). In other words, a good nanocavity for McOM should have an extinction cross section that is dominated by scattering. We finally note that for several applications it will be important to maximally collect the scattered field on a detector. The collected fraction of the scattered power is denoted by η_out_.

A subtlety that is probably unique to McOM is the existence of a quasi-continuum of dark plasmonic modes that may spectrally overlap with the bright mode of interest (for which all parameters above are computed). Sometime referred to as the “pseudomode” in the regime of strong coupling,^93,94^ this quasi-continuum is responsible for quenching the emission of a dipole placed in the near field; the β-factor is defined as the fraction of photons injected in the bright mode of interest vs the total photon emission rate in the near-field that includes all quenching channels.^83^ We explain how to compute β (which is a priori different for Stokes and anti-Stokes sidebands) in the Appendix. We recall that the β-factor is a useful figure-of-merit for single-photon sources embedded in photonic structures such as waveguides and micropillars, where it quantifies the likelihood that a photon is emitted in the guided mode vs the continuum of free-space modes and is intimately linked to the Purcell factor.^95^

The subtlety is that, in McOM, quenching by the quasi-continuum actually enhances the Raman scattering rate in the near field compared to the rate computed from the single-mode approximation presented above, by a factor 1/*β*; but the corresponding Raman photons are never detected (see Figure 3d). This “dark channel” for Raman scattering was invoked in recent observations of pronounced vibrational pumping while the detected Stokes scattering was much too low to compete with the expected vibrational relaxation rate.^47^ When computing *g*_0_ based on the full dyadic Green’s function calculation, however, there is no need to correct the resulting Raman scattering rate by 1/*β* since that approach already accounts for the total photonic local density of states at the position of the molecule.^74,75^ In that case, the collection efficiency–including quenching effects–is obtained from the computation of the Green’s function between the molecule and the detector position.^74^

## Nanocavity Examples

**Figure 4:**
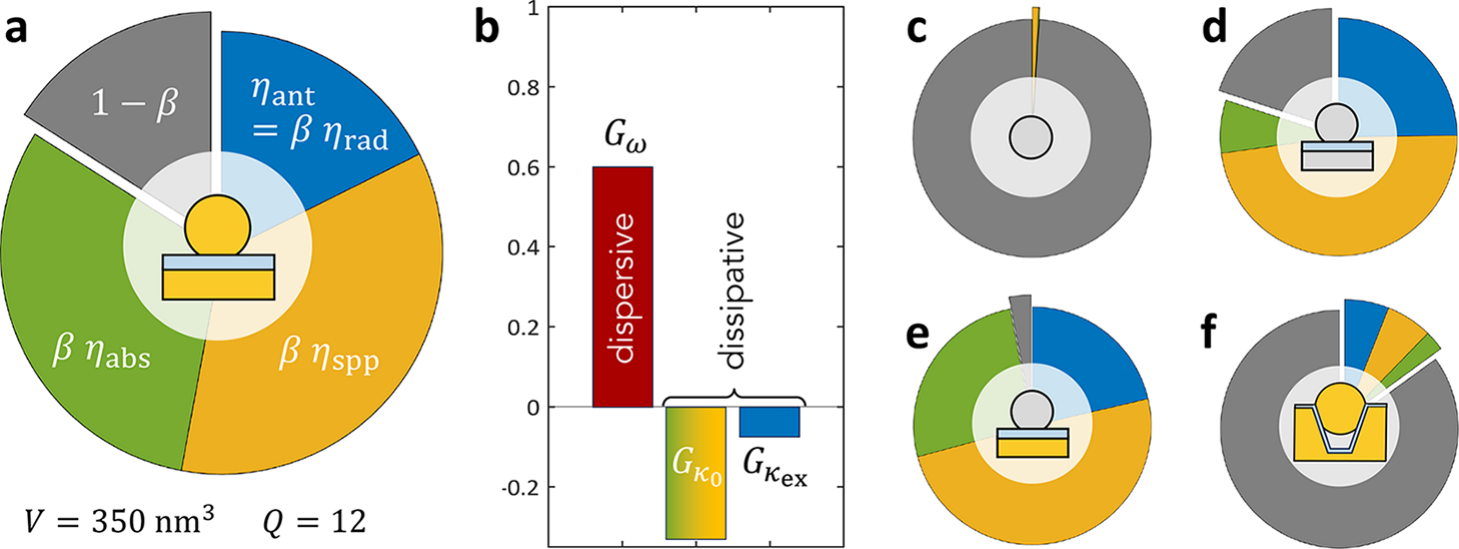
(a) Breakdown of decay channels for a dipole emitter optimally coupled to the bright mode of an NPoM cavity. The share 1 – β corresponds to quenching; it is not a modal quantity (but depends on wavelength and position of the emitter). Mode volume is computed at the position of highest dipolar coupling to the mode, and *Q* factor is evaluated by the spectral line width. Results were confirmed by QNM calculations. (b) Relative contributions of dispersive and dissipative coupling rates, estimated by varying the permittivity in the nanogap and computing its effect on ω_*p*_, κ_ex_ and κ_0_. (c–f) Breakdown of decay channels for the dominant bright mode of several nanostructures depicted as insets. A more complete list of parameters for various geometries is provided in Figure 6, Appendix.

To practically illustrate the general formalism we present quantitative results in Figure 4 computed for a few typical geometries, starting with a gold nanoparticle-on-mirror (NPoM) cavity on a gold substrate where the Raman active molecules form a monolayer acting as a nanometric spacer. We stress here that some values, such as the mode volume and quenching rate, depend on the emitter position, which is here chosen so as to minimize mode volume. Accurately treating the collective optomechanical coupling of the Raman-active monolayer requires more advanced approaches (e.g., based on the Green’s function as presented in ref (51)) and remains a topic for future research. We remark in Figure 4a that in spite of the nanometric distance between molecule and metal, the quenching of Raman emission is limited to 1 – β ≃ 16%, an attractive property of metallic nanogaps that has been discussed in refs (84, 87, 96−98) for example. The mode volume is 350 nm^3^, close to the physical volume between the gold mirror and the facet of the nanoparticle. With a *Q* factor of 12 at 760 nm, this dipolar bright mode decays into three channels: radiation (share ), internal losses by local absorption in the metal (η_abs_) and losses as surface plasmon polarition (SPP) emission (η_spp_), with . The share η_spp_ is here counted as a nonradiative loss channel, but proper engineering of the metallic substrate (e.g., with gratings^67^) may convert SPP into free-space radiation, in which case η_rad_ would increase accordingly, providing a substantial lever for improving radiative coupling efficiency in NPoMs. The input coupling efficiency depends on the excitation beam parameters, in particular its focusing and polarization; it is estimated as η_in_ ∼ 6% only, which should be taken as an upper bound for an optimally polarized and focused beam^86^ and in the absence of excitation through SPPs.

Figure 4b summarizes the relative magnitudes of dispersive and dissipative coupling rates. Beware that they should not be summed up to obtain a total optomechanical coupling rate since they have distinct impacts on the system’s dynamics.^99^ These results were obtained by varying the permittivity ε of the dielectric layer representing the molecules in the simulation and monitoring the effect on the resonance frequency and decay rates of the nanocavity mode of interest, with ,  and . This approach is simpler than the one employed in ref (90) where a single molecule was modeled by a dielectric ball of varying radius, but confirms that dissipative couplings are of similar magnitude as the dispersive coupling in McOM and should not be neglected (yet they have been in most works to date).

We present other examples of nanocavity geometries in Figures 4c-f. The single silver sphere antenna in Figure 4c was simulated using both COMSOL and analytical Mie theory, yielding very similar results (see also Figure 6). It is characterized by a bright dipolar mode having very good radiative efficiency η_rad_ ≃ 85%, yet presents a high quenching rate 1 – β = 99% (for a molecule 1 nm away from the sphere), illustrating the important distinction that should be made between the modal brightness η_rad_ and what is often called the antenna efficiency η_ant_ = *βη*_rad_ when quenching is a dominant loss channel (small β).^83^ Two other flavours of NPoMs are presented in Figures 4d,e to illustrate how the choice of metals may affect the shares of quenching and absorption. The last example in Figure 4f is the nanoparticle-in-groove used in ref (12) as dual resonant cavity for mid-infrared upconversion. This design suffers from dominant quenching, probably related to the large size of the nanoparticle (150 nm), calling for improvements. The full list of simulated parameters for these geometries is provided in the Appendix, Figure 6.

## Pico- and Nanocavities

A special class of systems to which the theory of McOM was employed are so-called picocavities, which are believed to be atomic-scale hotspots randomly created in laser-illuminated nanocavities during SERS measurements.^38^ Their phenomenology consists of a set of suddenly appearing new Raman peaks (the “picocavity signal”) under continuous excitation, whose fully correlated fluctuations point to a single-molecule origin. They coexist with the usual SERS peaks (the “nanocavity signal”) whose intensities generally remain weakly affected by the appearance of a picocavity. The observed linear increase of the anti-Stokes to Stokes intensity ratio of the picocavity peaks with laser power was consistent with the prediction of quantum backaction by the McOM theory.^38^ (As mentioned earlier, McOM provides a set of rate equations for quantum backaction that are fully equivalent to those for vibrational pumping in SERS.^60^) This observation implies that the cooperativity  is comparable to the thermal occupancy of the vibrational mode .

Under the assumptions used in ref (38) that the decay rates κ and γ and the intracavity photon number *n*_*p*_ are identical for nanocavity and picocavity, and with Raman polarizability estimated from DFT calculations of the bare molecule, the observation of vibrational pumping is consistent with an effective picocavity mode volume *V*_pico_ on the order of 1 nm^3^. Such values, which are two to 3 orders of magnitude smaller than those of nanocavities, have been also predicted by electromagnetic simulations^100−^^103103^ (Figure 4). We stress here that McOM is not a theory intended to explain the formation of picocavities.

If we now apply the scaling law presented above, which states that the cooperativity scales as *Q*/*V* in McOM for filled cavities, we arrive at the intriguing prediction that the Raman signal from a picocavity could be 2 or 3 orders of magnitude stronger than the stable nanocavity signal. We emphasize here that this prediction already accounts for the fact that hundreds of molecules contribute to the nanocavity signal whereas just one does for the picocavity signal. Such giant Raman intensity enhancement has so far not been observed: experiments reported picocavity signals of similar magnitude as the nanocavity signal, which itself shows little modification during a picocavity event.^38,102−108^ There could be multiple explanations for this apparent discrepancy: On the one hand, molecules in picocavities could possibly suffer from stronger quenching, reducing η_rad_. On the other hand, a chemical enhancement mechanism could play a dominant role in explaining picocavity spectra, as suggested by DFT calculations that include just a few gold atoms around the molecule.^107^ Finally, it is believed that large field gradients at the scale of the molecule are an essential feature of picocavities,^38^ so that this type of coupling should be properly integrated in the McOM model, originally built around the dipole approximation. In any case, it would be a worthwhile undertaking to gain better understanding of all parameters that enter the expression of detected Raman photon flux in such systems (Figure 3).

## Experimental Challenges

Multiple of the physical phenomena predicted by the McOM theory await solid establishment in experimental demonstrations. To understand the challenges, we should first detail the main experimental requirements for rigorously applying the McOM formalism and test its predictions. A first set concerns the knowledge of the system:

- Single nanocavity measurements are to be privileged; if measuring an ensemble one must be certain that all nanocavities and their molecular contents are absolutely identical (which is difficult in practice given the nanometric critical dimensions involved).
- The optical response of the nanocavity should be fully known in the frequency region covering the laser and Raman sideband. Experimentally, dark-field scattering spectroscopy provides valuable information at the single particle level, yet it is only sensitive to bright modes that efficiently scatter an incoming plane wave and does not suffice to reconstruct the near-field response that also depends on dark modes. It is also not able to distinguish the contributions from κ_ex_ and κ_0_ to the observed *Q*-factor. Numerical simulations can be helpful to obtain the decay rates κ_ex_, κ_0_, the mode volume *V* and the bright mode branching ratio β. Simulations are quite accurate for gaps larger than 1 nm but require a precise knowledge of the geometry and refractive index of the molecular spacer (for gap nanocavities). Usually, these parameters are fine-tuned in the simulations to reproduce the measured dark-field scattering spectra.^109^ Electron beam methods offer nanoscale insights into the plasmonic modes,^110^ but NPoM geometries and vertical nanogaps in general are not readily accessible.
- To obtain the coupling rate *g*_0_ and single-photon cooperativity , knowledge of the Raman polarizability and the exact number of embedded molecules is required. In reality, each molecule has a different coupling rate depending on its exact location due to the inhomogeneity of the near-field, which can be treated numerically.^51^ But the main source of uncertainty here is the single-molecule Raman polarizability, and more particularly, how much it is affected by “chemical” enhancement for molecules bound to the metal.^45,111−113^ Accurate calculations of Raman cross sections for large enough metal clusters coupled to small organic molecules remain difficult and they suggest that the chemical enhancement factor is usually large.^107^ Alternatively, a more controllable thin material such as graphene^114^ could be used as an intermediate intermediate layer between the plasmonic metal and the molecules, in an attempt to better separate chemical and electromagnetic enhancements.
- Last, but not least, the intracavity plasmon number *n*_*p*_ must be estimated for a given pump power, which requires the additional knowledge of η_in_. Focused cylindrical vectorial beams allow optimizing the power coupled to specific nanocavity modes,^67,86^ but a quantitative knowledge of η_in_ remains elusive. Obtaining this parameter from a simulation implies the use of a focused beam as background field, often much harder to implement than a plane wave excitation,^115^ in particular for nanocavities built on mirrors.^116^ Experimental measurements of η_in_ or *n*_*p*_ are challenging in the context of McOM and SERS. Interferometric scattering microscopy^117^ was successfully employed to quantify the extinction of nanoantennas on glass^118,119^ and should therefore allow retrieving η_in_ in the context of McOM, where most cavities are assembled on a mirror substrate, though. Alternatively, and as elaborated below, the experimental geometry can be modified to resemble more that of the simulated plane wave excitation.

In addition to these challenges linked with the difficulties to fully characterize the near-field response of a gap nanocavity, another hurdle in the quest for McOM effects and applications is the limited tolerance of metallic nanocavities to large excitation powers. In ref (51), for example, irreversible changes to the Raman spectra (which can be associated with optical “damage” in this context, Figure S23 in ref (51)) represent a non-negligible portion of the studied power-dependent effect–even though much care was taken to limit this damage by short laser exposure time and optimized pulse duration. The most exciting predictions of McOM are realized in the regime where , so that the intracavity plasmon number *n*_*p*_ must reach the largest possible values–at least be of order unity. To avoid excessive heating, this regime has been mostly explored under picosecond pulsed excitation.^46−49,51^ This causes at least two types of difficulties. First, because η_in_ is usually much smaller than one, most of the incoming laser power is not even coupling to the nanocavity mode of interest, but it can nevertheless be absorbed in the focal area and generate unwanted heat, hot-electrons, etc. Second, as *n*_*p*_ increases inside the nanocavity, other nonlinearities may emerge and are hard to experimentally distinguish from potential optomechanical effects. In particular, both thermal effects^109,120,121^ and electrons excited in the metal^122−125^ are expected to modify the transient plasmonic response on time scales down to picoseconds^126−128^ – comparable to the vibrational decay rate that dictates the optomechanical response.

In fact, it remains an open question whether optomechanical nonlinearities can be observed before the onset of plasmonic nonlinearities. In most studies of ultrafast plasmonics on metallic nanoparticles, measurable nonlinear response (such as resonance shift and broadening) is observed for pump fluences as low as 100 μJ/cm^2^ at off-resonant near-infrared wavelengths (see for example refs^125, 126, 128^, among others). When using a high pulse repetition rate of ∼100 MHz typical of Ti:Sapph oscillators, this fluence translates in an average power of 100 μW/μm^2^. Considering a diffraction-limited spot-size in a confocal microscope with numerical aperture of 0.8–0.9, and that excitation is usually resonant with a plasmonic mode in McOM experiments, it means that few tens of microwatts–or even less–averaged power may be enough to drive a significant nonlinearity in the plasmonic response. In comparison, the highest average power used in ref (51) was 60 μW. The optomechanical effects being investigated should therefore be carefully deconvoluted from the nonlinear plasmonic response itself, motivating further work.

## Theoretical Challenges

- In contrast with macroscopic and mesoscopic mechanical resonators, molecular vibrations feature markedly anharmonic potentials, in particular for modes that are localized to few molecular bonds.^58^ The standard McOM Hamiltonian, however, assumes a harmonic potential for the vibration. One recent theoretical work extends the formalism to account for vibrational anharmonicity in the case of a single, off-resonant molecule.^129^ When sufficiently sharp features are present in the LDOS of the nanocavity, this work predicts a regime of incoherent mechanical blockade, provided that only the Stokes transition to the first excited vibration can be efficiently pumped by SERS, while the second one is not. Such sharply changing LDOS can be achieved for example by the Fano line shape of a hybrid plasmonic-dielectric cavity.^130,131^ Another finding of ref (129) is that anharmonicity has deep impacts on the regime of dynamical backaction amplification: it changes the threshold for coherent mechanical oscillations and lowers their amplitudes. A recent proposal also suggests that vibrational anharmonicity is a resource in single-molecule optomechanics for the production of antibunched photons through optomechanical blockade.^132^ It is likely, however, that such effects rapidly disappear in the experimentally relevant setting of many molecules, since the collective modes should become harmonic.^132^ Solving a model that includes anharmonicity and collective effects remains an open challenge. We also mention here the case of strongly driven picocavities, where vibrational pumping combined with anharmonicity can lead to qualitatively similar Raman peak shifts as predicted from the optical spring calculation.^51^
- Primo et al. put forward that dissipative optomechanical coupling can be on par with dispersive coupling in McOM.^90^ Dissipative coupling occurs when the mechanical displacement modifies one of the dissipation rates of the cavity (κ_ex_ and/or κ_0_).^99,133^ The formula for *g*_0_ originally derived in refs (5, 7) does not capture dissipative coupling, which is intimately related with the multimode and nonhermitian nature of nanocavities.^134^ The presence of dissipative coupling impacts the shape and magnitude of the optomechanical damping rate vs laser wavelength.^99,133^ Ref (90) applies perturbation theory in the quasi-normal mode formalism to compute an imaginary part of the optomechanical coupling rate that corresponds to dissipative coupling, confirming the results with full-wave moving-mesh simulations. They conclude that dissipative coupling is at least of comparable magnitude as dispersive coupling in the canonical NPoM nanocavity, which we confirm using a simplified approach in Figure 4b. It is also expected to prominently feature in engineered multimode McOM systems, like hybrid nanoantenna-cavity resonators.^73^

A point that has not been explicitly clarified in the literature to date is whether the Green’s function approach to McOM,^74,75^ that can treat arbitrary plasmonic and dielectric resonators, implicitly accounts for dissipative coupling. The difficulty here is that the Green’s function approach does not yield an optomechanical coupling rate *g*_0_ but directly provides instead expressions for the dynamics of vibrational and plasmonic operators.

- Resonant and near-resonant Raman scattering could in first approximation be thought of as just increasing the Raman polarizability that enters the McOM model desribed in the first part. But recent theoretical work by M. Martinez-Garcia et al. concluded that, even in the weak optomechanical coupling regime, interference between resonant and nonresonant contributions to SERS can deeply alter the predicted nanocavity scattering spectrum.^135^ Previously, S. Hughes et al. already investigated the quantum nonlinear regime of resonant strong coupling between the nanocavity mode and the molecular electronic transition, in the presence of vibrational modes.^136^ Future experimental and theoretical works are needed clarify the precise impact of molecular electronic resonance on vibrational dynamics and nanocavity scattering spectrum in the context of accessible SERS scenarios.
- As already discussed above, experimentally determining the intracavity plasmon number remains an open challenge; conversely, numerical estimates are usually performed under the simplifying assumption of plane wave excitation and there is little quantitative work that accounts for strongly focused laser beams, possibly with complex vectorial fields.^86^ Such simulations do exist^115,116^ but have not been implemented yet in the context of McOM to obtain more accurate estimates of input coupling efficiency and intracavity plasmon number.
- A resource-efficient formalism that correctly accounts for collective effects among molecules coupled to a same nanocavity is still missing. The pioneering calculations presented in ref (51) require numerical evaluation using a discrete set of point dipoles representing the molecules, limiting this approach to 100 molecules so far, while typical nanocavities can host 1000 or more. Since 2D materials can also be used as Raman active layers in McOM,^48,49^ the development of a continuum phonon model that applies also for molecular layers and accounts for collective dynamics would be valuable. Additionally, it was recently proposed that dipole–dipole coupling among closely packed IR-active molecules is an important source of collective behavior^137,138^ having impacts on the Raman spectra;^139^ it would be appealing to include such effects in a comprehensive McOM description.

## Molecular Optomechanically Induced Transparency

Contrary to macroscopic cavity optomechanics, where heterodyne linear optical field measurements are the norm, McOM has been so far only studied with photon-counting detectors such as CCD cameras, possibly losing valuable phase information in the process. In particular, depending on the detuning between laser and cavity resonance the optomechanical (Raman) sidebands are predicted to consist of different contents of phase and amplitude modulations: Pure phase modulation is expected on resonance, which can be readout by homodyne or heterodyne detection. We therefore suggest to introduce the use of local oscillators generated by a nonlinear optical process in the measurement of SERS signals from nanocavities, as already implemented in the case of stimulated Raman scattering (SRS) imaging^141^ and vibrational sum frequency generation.^36^

**Figure 5:**
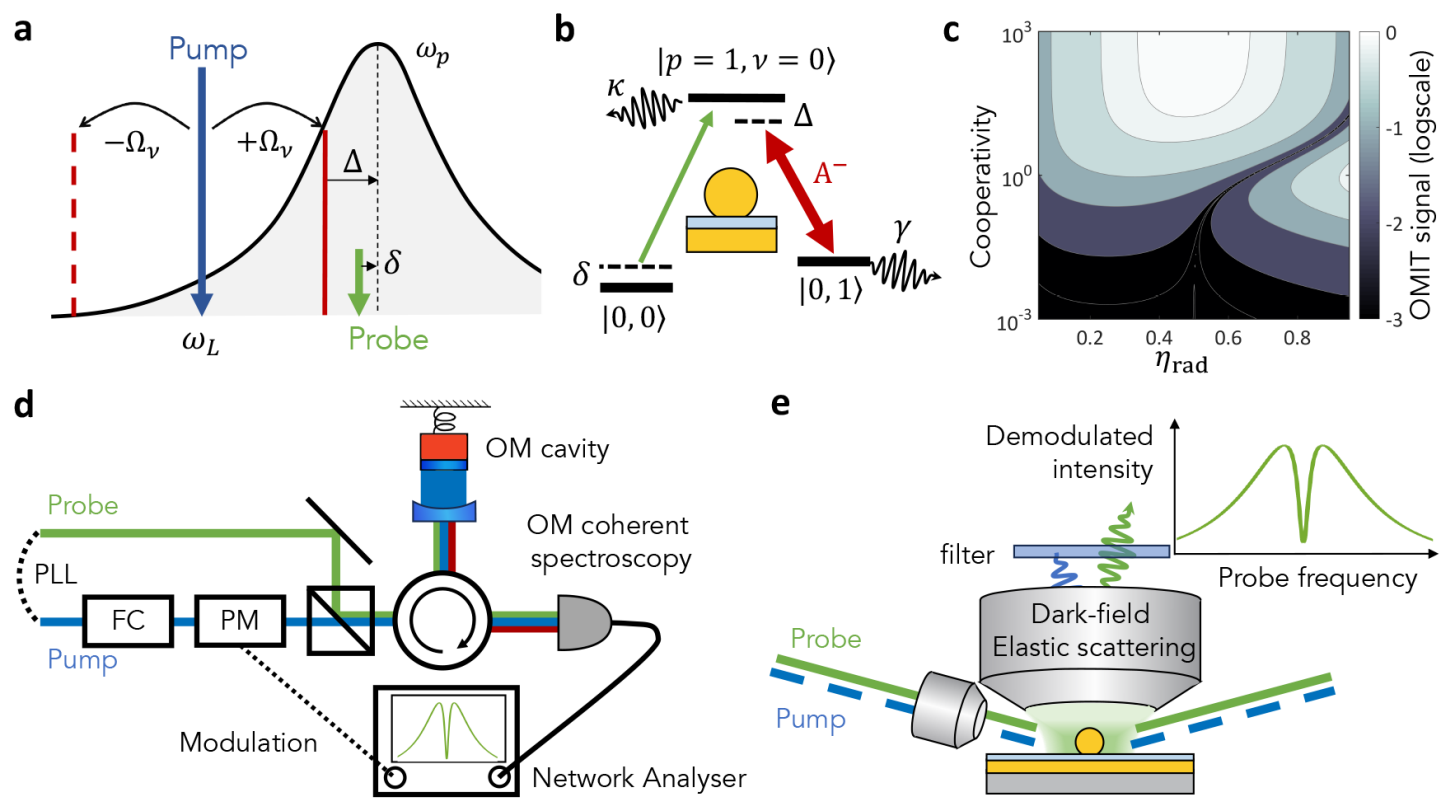
Optomechanically induced transparency: concept and measurement schemes. (a) Frequency representation of all involved fields and resonances with the definitions of detunings Δ and δ. (b) Same parameters represented on an energy level diagram in the single-excitation subspace. The notation |*p*, ν⟩ designates a state with *p* plasmons and ν vibrations. (c) Computed map of OMIT signal strength (normalized to the input probe power) vs cooperativity  and external coupling efficiency η_rad_. (d) Possible OMIT measurement scheme in macroscopic cOM. Adapted with permission from ref (140). Copyright 2020 American Physical Society. PLL, phase-locked loop; FC, filter cavity; PM, phase modulator. (e) Proposed implementation of OMIT measurement in McOM using a dark-field geometry for pump and probe excitation. A spectral filter blocks the pump, and the probe signal is sent to the detector. The pump (pulsed or cw) is intensity-modulated so that the demodulated scattered probe intensity senses the pump-induced change in cavity scattering, which can have thermal (broad plasmonic response) or optomechanical (narrow vibrational response) origins.

**Figure 6:**
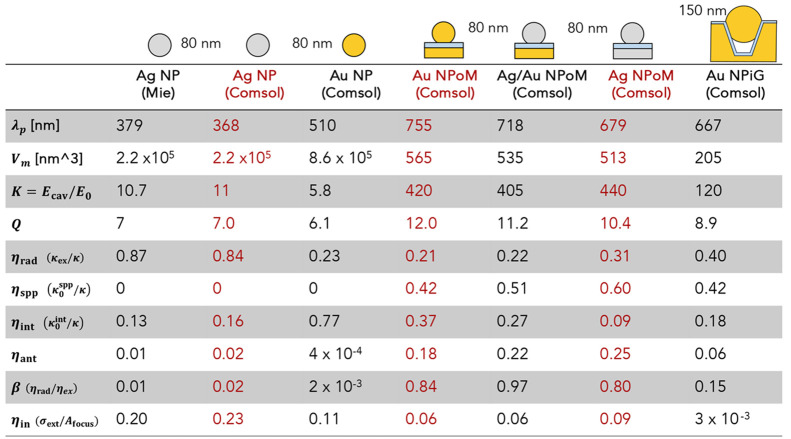
Full list of parameters extracted from the simulations of various plasmonic nanostructures. The parameters characterize the dipolar plasmonic mode for the single NP cases, the L01 mode for the NPoM cases, and the antisymmetric L01 mode for the NP-in-groove (NPiG) case. *E*_cav_ is here defined as the spatial maximum of the norm of the electric field at a distance *d* = 1 nm from the nanoparticle’s surface, and *E*_0_ is the norm of the background electric field generated or by a plane-wave excitation or by a point dipole source. For nanogap structures (NPoM and NPiG), *E*_cav_ is taken on the midgap plane, which is at a distance of 0.65 nm from the surface considering a single layer of molecules 1.3 nm thick. To calculate *A*_focus_, a focusing objective of numerical aperture NA = 0.9 is considered. The NPs are modeled by a sphere of 80 nm diameter. The NPoMs are composed by a truncated sphere of 80 nm with a facet size of 10 nm and a 300 nm thick metallic film separated from the sphere by a dielectric layer (1.3 nm thickness, refractive index of 1.4). For the NPiG case, the dimensions of the truncated sphere are modified (150 nm diameter without facets), and the groove dimensions considered are as follows: 1.4 μm long with a trapezoidal cross section whose bases are 180 and 80 nm; the height is 150 nm. The corners are rounded by 20 nm. For plane-wave excitation simulations of the nanogap structures, we consider an incident wave at an angle of 60 deg from the gold plane’s normal (p-polarized for the NPoM case, orthogonal to the groove for the NPiG case).

Related, a valuable measurement that was not yet implemented as such in McOM is that of optomechanically induced transparency, or OMIT.^142,143^ Beyond its fundamental appeal and potential applications,^144^ OMIT measurements allow a very direct estimate of the cooperativity, a capability that is still lacking in McOM. The effect is described in Figure 5a,b: a strong pump laser is tuned on the lower vibrational sideband of the cavity, so that the anti-Stokes frequency is near resonant with the cavity frequency (their detuning is denoted as Δ). A weaker probe laser beam is swept in frequency (detuning δ from the cavity). In macroscopic cOM, a destructive interference between the two excitation pathways depicted in Figure 5b can induce a transparency window for the probe when Δ ≃ δ compared to its absorption by the cavity in the absence of pump field. A detailed calculation adapted to the specificity of McOM (where scattered light is detected instead of reflection or transmission) has not been published to our knowledge; however we expect that a sharp feature is expected in the probe scattering spectrum (peak or dip) at Δ ≃ δ. Panel (c) in Figure 5 estimates the relative strength of this feature (for the case Δ = 0) as a function of cooperativity  (where *n*_*p*_ scales linearly with pump power) and external coupling efficiency κ_ex_/κ = η_rad_. Two regimes are favorable to the observation of OMIT: (i) overcoupled cavity (η_rad_ ∼ 1) with moderate cooperativity , or more conventionally (ii) high cooperativity  under critical coupling (η_rad_ = 0.5).

A typical OMIT measurement scheme in macroscopic cOM is presented in Figure 5d; how should OMIT be measured in the context of McOM on a plasmonic nanocavity? The first challenge is that plasmonic nanocavities are not probed in reflection or transmission given their subwavelength dimensions, but rather in scattering geometry. What matters is to isolate the optical field that has interacted with the nanocavity mode. For that, we propose a dark-field scattering detection geometry (Figure 5e) where the specular reflection of pump and probe beam are not collected. The pump frequency is filtered out of scattered field and the probe power is monitored; modulating the pump intensity and demodulating the probe signal using lock-in detection should allow to reach the required sensitivity for observing OMIT with state-of-the-art nanocavities (Figure 5c). Compared to recent implementations of SRS on plasmonic antennas,^145,146^ the proposed scheme mainly differs in the dark-field detection geometry and the prerequisite to be able to observe the absorption or scattering of a single nanocavity; also, note that the pump and probe beams in OMIT correspond to the Stokes and pump beams in SRS, respectively. This measurement could be performed under ps pulsed or continuous wave excitation.

## Conclusion

By reformulating the physics of SERS in the language of cavity optomechanics, the framework of molecular cavity optomechanics opens a new arena for understanding fundamental aspects of plasmon-enhanced Raman scattering and for applying this phenomenon to nonlinear nano-optics and possibly quantum technologies. In this Perspective, we first clarified the content of this framework and how to apply it to typical nanocavities used for SERS. We identified the need for a better characterization of the input coupling efficiency and different dissipation channels of the nanocavity field, and recalled that the strength of optomechanical effects should be quantified by the cooperativity instead of the coupling rate alone. The methodology for calculating these quantities was illustrated on a couple of concrete and relevant examples, and a partial overview of nanocavity performance compared to dielectric microcavities was proposed. A number of experimental challenges were listed, which still prevent unambiguous identification of and control over predicted optomechanical effects in SERS such as dynamical backaction amplification, optical spring, or collective vibrational response of molecular ensembles. Some fundamental questions remain open at the level of the theory itself as well, such as how to include vibrational anharmonicity, how to properly account for dissipative coupling, or how to formulate a continuum model of the vibrational mode for many molecules or 2D materials.

Looking ahead, we can see numerous promising and challenging research directions. Hybrid plasmonic-dielectric approaches may allow to reach overcoupled cavities as well as deeply sideband-resolved McOM. New ideas are needed to prevent metal surface restructuring under high laser power, and the failure mode under pulsed excitation should be clarified. Molecules with long-lived vibrational modes and large Raman cross-section should be engineered to boost the cooperativity. Entanglement between plasmon and molecular vibrations, and among vibrating molecules, should be demonstrable. Exploiting resonant or near-resonant coupling to electronic molecular transitions would establish tripartite phonon-photon-exciton systems,^136,147−151^ bearing analogy to macroscopic optomechanical systems that include two-level systems, with potential for enhanced coupling strengths, nonlinear effects, and applications in wavelength conversion.^152,153^ Finally, the realization of the single-photon optomechanical strong coupling would give rise to a nonlinear response at the quantum level, both for photonic and phononic degrees of freedom, a holy grail in quantum optomechanics.^154,155^

## Figures and Tables

**Figure 7:**
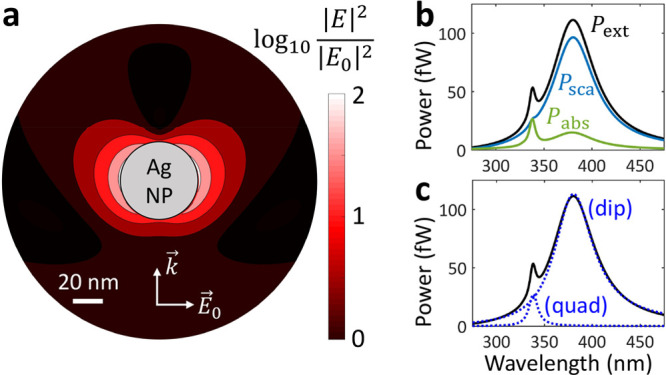
Mie calculations for a spherical silver NP of 80 nm diameter under plane-wave excitation. (a) Spatial distribution of the intensity enhancement factor around the silver NP for a plane-wave excitation tuned to the dipole resonance. (b) Spectra of extinguished, scattered and absorbed power. (c) Multi-Lorentzian fit of the extinguished power with the dipolar and quadrupolar contributions labeled (dip) and (quad), respectively.

## Appendix

This appendix provides additional guidelines regarding the calculation of the cavity parameters introduced in this Perspective. It has to be noted that many aspects regarding calculations of cavity parameters for plasmonic cavities have been covered in depth elsewhere.^5,7,45,83,87^ Below, we focus on a simple case study (a small spherical nanoparticle, NP) for which a Mie theory approach is possible. The spherical NP case, being extensively investigated in the literature, constitutes the most elemental way to introduce cavity parameters for the plasmonics community. It also offers a natural benchmark to evaluate and connect the different frameworks existing in the literature. Building on the NP case, we conclude the Appendix with a presentation of the FEM simulations used to address more complex structures, such as the NPoM and NP-in-groove (NPiG) geometries. Some representative results are summarized in Figure 6.

### Mie Approach: Spherical Nanoparticle

The Mie theory gives an exact solution to the problem of the scattering of an electromagnetic (EM) wave by a sphere. In addition to the EM field solutions at any point in space, this theory conveniently provides analytical expressions for integral quantities such as the scattered power *P*_sca_ and the absorbed power *P*_abs_ (see Figure 7). For a sufficiently small silver nanosphere, all parameters of the plasmonic dipole mode can be accurately described using scattering expressions. Using a Mie solver package,^1^ the spectra *P*_sca_(λ), *P*_abs_(λ) and *P*_ext_(λ) = *P*_sca_(λ) + *P*_abs_(λ) are obtained by scanning the wavelength of the plane-wave excitation (PWE) at constant incident intensity (*S*_inc_ = *P*_inc_/*A*_focus_, with *A*_focus_ the effective focal area of the incoming light away from the nanoparticle). Analytical expression of *A*_focus_ for different light waves and its connection with the normalized energy density of the wave, can be found in ref (156).

A multi-Lorentzian fit of *P*_ext_(λ) determines the dipole resonance wavelength (λ_p_) and its decay rate (κ). Other single-mode cavity parameters are subsequently derived from direct evaluation of the Mie calculation results on resonance: the effective cross-section σ_ext_(λ_p_) = *P*_ext_(λ_p_)/*S*_inc_, the radiative efficiency η_rad_ ≈ *P*_sca_(λ_p_)/*P*_ext_(λ_p_), the non-radiative efficiency η_abs_ ≈ *P*_abs_(λ_p_)/*P*_ext_(λ_p_) and the local field enhancement factor *K*(*d*) at a distance *d* from the nanoparticle’s surface. Implicitly, by applying a multi-Lorentzian fit (Fig. 7c), we assume here that the multimode *P*_sca/abs_ can be decomposed into its single-mode components. To quantify the impact of other plasmonic modes on a point-like object at a distance *d* from the NP’s surface, we make use of Mie calculations with a point-dipole excitation (PDE). The (total and radiative) Purcell factors extracted from the PDE calculations, *M*_tot_ and *M*_rad_ respectively, enable to evaluate the antenna efficiency: η_ant_ = *M*_rad_/*M*_tot_. Since all plasmonic modes contribute to *M*_tot_, the ratio β = η_ant_/η_rad_ quantifies the probability for the dipole to emit into the bright mode; the probability of emission into other modes, i.e. quenching is then given by 1 – β.

The remaining single-mode cavity parameters can be calculated from the following expressions.

- The mode volume *V* ≃ σ_ext_*c*/(*K*^2^κ),^5^ with *c* the speed of light in vacuum.
- The mean intracavity photon number *n*_*p*_ under laser illumination of power *P*_inc_ at frequency ω_*L*_/(2π) detuned from the cavity resonance by Δ = ω_*L*_ – ω_*p*_ = ω_*L*_ – 2*πc*/λ_*p*_:^2^

with η_in_ = σ_ext_/*A*_focus_ the input coupling efficiency.

This simple approach can be applied to more elaborated structures via FEM and FDTD simulations as long as the targeted mode is sufficiently well-isolated (spectrally or spatially from the contributions of other modes).

### Estimation of the Mode Volume *V*: Alternative Method

The total Purcell factor *M*_tot_ calculated from the PDE calculations is dominated by a continuum of modes^93^ even at the dipolar mode’s resonance frequency so that direct mode volume calculation would not be possible. On the contrary, for a PDE tuned in resonance with a sufficiently isolated cavity mode, the radiative part of the Purcell factor *M*_rad_ is, to a very large extent, due to this mode only. With the help of the efficiencies calculated previously, we can approximate the total Purcell factor due to a single dipolar bright mode as *M*_tot_^(dip)^=*M*_rad_(1+η_abs_/η_rad_)=*M*_rad_(1+κ_0_/κ_rad_). This factor can be used directly to approximate the mode volume following the approach of ref (7) or be linked to the approach exposed above via the following relation between Purcell and field enhancement factors: *K*^2^=η_rad_*M*_tot_^(dip)^. It has to be noted that both mode volume estimates given here are in very good agreements with the values found by F. Koenderink in ref (157) for small spherical Ag NP and with our own evaluations via FEM calculations of the mode volume following a quasi-normal mode (QNM) treatment.^83^ A similar level of agreement is found for the other cavity parameters calculated for the single NP case.

### Beyond the Mie Theory Approach

A rigorous modal treatment, based on the quasi-normal mode formalism, is possible for many of the parameters introduced above.^83^ The direct evaluation of η_rad_, named modal brightness  in the QNM framework, remains however a tedious task. Indirect calculations of η_rad_ for NPoM cavities have been introduced in ref (87). Performing PDE calculations followed by near-to-far field transformation (NFFT),^158^ the energy dissipated through radiative and guided-mode (SPP) channels can be separated. This way, η_ant_ and *βη*_spp_ are found. Additional QNM calculations enable to evaluate β so that the multimode absorption channel *βη*_abs_ = β – η_ant_ – *βη*_spp_ as well as all single-mode counterparts {η_rad_, η_spp_, η_abs_} can be quantified.

In this Perspective, and in analogy with the single NP treatment discussed above, we perform PWE calculations on resonance with the plasmonic mode of interest followed by NFFT to evaluate the single-mode loss channels η_rad_ and η_spp_. Similarly to the treatment of ref (157), we evaluate the divergence of the Ohmic losses with the size of the integration domain^159^ to separate the SPP contribution to absorption from the single-mode localized absorption (η_abs_).
